# Prevalence of patients with limited health literacy in rotator cuff tears and their experiences with care: a mixed-methods study

**DOI:** 10.1016/j.jseint.2026.101642

**Published:** 2026-01-28

**Authors:** Evy E.J. Jetten, Frederik O. Lambers Heerspink, Isabella C. Klarenbeek, Rosaline Mentink, Taco Gosens, Esther R.C. Janssen

**Affiliations:** a Department of Orthopaedic Surgery, VieCuri Medical Centre, Venlo, The Netherlands; b Department of Orthopaedics and Research School Caphri, Maastricht University Medical Centre, Maastricht, The Netherlands; c Pharos, Dutch Centre of Expertise on Health Disparities, Utrecht, The Netherlands; d Department of Orthopaedics, Elisabeth Hospital (ETZ), Tilburg, The Netherlands; e Department of Medical and Clinical Psychology, Tilburg University, Tilburg, The Netherlands; f IQ Health Science Department, Radboudumc, Nijmegen, The Netherlands; g Academie Paramedische Studies, Hogeschool Arnhem en Nijmegen (HAN), Nijmegen, The Netherlands

**Keywords:** Health literacy, Rotator cuff tear, Patient education, Health care communication, Patient challenges, Health care information, Health literacy screening

## Abstract

**Background:**

Limited health literacy (LHL) can affect the ability to understand and apply health information. For patients with rotator cuff tears, following post-treatment guidelines is crucial for recovery. However, existing resources often don't meet the needs of those with LHL, potentially delaying recovery. This study investigates the prevalence of LHL in patients with rotator cuff tears, the challenges in managing health information, and possible strategies to improve care.

**Methods:**

The prevalence of LHL in patients with rotator cuff tears was assessed using the Single-Item Literacy Screener. To explore the challenges in managing health information and possible solutions, semistructured interviews based on the Health Literacy Questionnaire were conducted with twelve patients. Data were collected through audio recordings and field notes, which were transcribed and analyzed using thematic analysis.

**Results:**

The prevalence of LHL in patients contacted by phone was 9.3% and 40.7% when contacted at the outpatient clinic. Patients experienced significant language barriers in health care communication, challenges in finding and evaluating relevant health information. These patients reported diverse and specific needs for tailored health information. They suggested using formats that cater to their preferences and sought support from their social networks to better manage their condition and treatment journey.

**Conclusions:**

This study adds to the evidence that LHL is common and associated with multiple challenges in health care engagement. Addressing these challenges through personalized, accessible communication strategies, in combination with support from their social environment, should be a priority for health care systems aiming to reduce inequalities and improve patient outcomes in patients with rotator cuff tears.

In Europe, the prevalence of limited health literacy (LHL) varies between 25% and 72% in adult populations.^16^ People with LHL experience difficulties in completing tasks related to the management of health related information and communication. This negatively impacts their ability to make health-related decisions, taking care of oneself, and access appropriate health care.^16^ As a result, LHL increases the risk of poor physical and mental health, and a higher likelihood of chronic conditions, such as type 2 diabetes, cancer, and osteoarthritis, increasing the risk of premature death.^3^^,^^27^ People with LHL use health care services more frequently, contributing disproportionately to health care costs compared to people with average health literacy.^27^ Certain circumstances can influence health literacy, for example, everyone may temporarily have LHL skills in high-stress situations.^36^

In orthopedics, little is known about the impact of health literacy on treatment outcomes. Limited evidence suggests there may be a relationship between health literacy and outcomes after orthopedic prosthetic procedures.^15^ However, for most orthopedic treatments, such as the treatment for a rotator cuff tear, the impact of health literacy on treatment outcomes is unknown. For patients undergoing rotator cuff repair treatment understanding and adhering to lifestyle and rehabilitation guidelines is crucial to ensure smooth recovery and preventing complications.^5^ This is especially relevant, as hospital stays are short and recovery primarily occurs at home. Successful rehabilitation therefore depends heavily on the patient's health literacy. Although numerous strategies for clear communication and health literacy have been proposed,^5^ research indicates that these recommended practices are often implemented inconsistently.^11^

Therefore, this mixed-methods study aims to 1) assess the prevalence of health literacy among patients with rotator cuff tears, 2) identify specific challenges encountered within the hospital care pathway by individuals with LHL, and 3) explore potential strategies to mitigate these challenges and enhance patient-centered care. We hypothesize that the prevalence of LHL of patients with rotator cuff tears is 25%.

## Methods

### Study design

This study used a mixed-methods design, utilizing a questionnaire to explore the prevalence of LHL and semistructured interviews to investigate challenges in and solutions to communication in the health care pathway of people with rotator cuff tears. Ethical approval for this study was obtained from the medical ethics review committee of azM/University Maastricht (reference number 2023-0317). The results are reported in accordance with the Mixed Methods Reporting in Rehabilitation & Health Sciences checklist.^34^

### Study population

Adult patients undergoing either elective rotator cuff repair surgery or conservative treatment for a full thickness rotator cuff tear at VieCuri Medical Centre in the Netherlands were recruited. In the region of Northern and Middle Limburg, the demand for health care and support is increasing at a faster pace and with greater complexity compared to other Dutch regions.^17^ Projections indicate that by 2040, the proportion of residents aged 65 years and older will rise from 24.5% to 31%, whereas the national average is expected to reach 25%.^17^ The region is further characterized by a rise in social problems, and relatively low socioeconomic status compared to other regions.^17^ To participate, patients were required to have sufficient proficiency in spoken Dutch or access to an interpreter who could assist with translation. Between April 2024 and May 2024, patients were contacted either by telephone or recruited in person at the orthopedic outpatient clinic, during which the Single-Item Literacy Screener (SILS) was administered. Between April 2024 and February 2025, patients with LHL identified in the prevalence study were recruited for the interview. Upon receiving written consent, the researcher scheduled semistructured interviews. After conducting 10 interviews, theoretical sampling was applied because no patients with non-Caucasian ethnicity were included and there was a relatively low number of women in the sample. Therefore, additional patients fitting these criteria were recruited to ensure a more diverse and representative sample.

### Data collection

The SILS was used to establish prevalence of LHL.^19^ The SILS is shown to be a valid measure of health literacy, as it correlates with more complicated, validated measures.^2^^,^^4^ The SILS shows acceptable diagnostic accuracy, yielding a sensitivity of 66% and specificity of 58% when compared with the comprehensive HLS-EU-Q16 with an area under the curve of 0.66.^33^ It evaluates a patient's need for assistance in reading written health information, thereby enabling an estimate of health literacy levels.

During the interviews data were collected using a semistructured interview guide. Interviews took place either at their home or before or after a scheduled appointment at the hospital. The interview guide was based on the nine domains of the Health Literacy Questionnaire: feeling understood and supported by health care providers, having sufficient information to manage my health, actively managing my health, social support for health, appraisal of health information, ability to actively engage with health care providers, navigating the health care system, ability to find good health information, and understand health information well enough to know what to do.^23^ Additionally, patients were asked to review health information provided during their treatment process (eg, appointment invitation and informational brochures) and identify areas where they experienced difficulties. Patients were also asked to discuss alternative methods of information delivery that could help them make informed health decisions during their treatment. The following demographic data were collected to characterize the study population: age, sex, ethnic background, household composition, comorbidities, and type of treatment.

### Data analysis

Descriptive statistics (means and standard deviations) were used to summarize the demographic characteristics of the study population. Differences in demographic characteristics between patients with limited and adequate health literacy were assessed using an independent *t*-test for normally distributed continuous variables or a Mann–Whitney *U* test for non-normally distributed continuous variables, and a chi-squared test for nominal variables. The prevalence of LHL within the population was calculated based on SILS outcomes. A minimum of 100 patients undergoing treatment for rotator cuff tears were necessary to calculate a prevalence of 25% with an α level of 5%, prevalence can be estimated with a precision of ±10%.^20^^,^^26^

For the interviews, audio recordings of the interviews and field notes were anonymously and verbatim transcribed. The transcripts were fragmented and open-coded using ATLAS.ti version 9^30^ (ATLAS.ti, Berlin, Germany). The open codes were further categorized into subthemes and themes through thematic analysis. The semistructured interviews were independently fragmented, coded, and thematically analyzed by two researchers (E.J.E. and I.K.). Discussions continued until a consensus on the identified themes was reached, which formed the basis for coding the remaining transcripts. Researchers had no established relationships with patients prior to the interviews. To ensure reflexivity, the team engaged in regular discussions to reflect on potential biases and their influence on data collection and analysis. A third researcher (E.J.A.) was consulted to resolve issues in case of disagreements, to refine themes, and to optimize the coding tree.

## Results

### Participant characteristics

A total of 147 participants were included in this study. The SILS was administered to 145 participants: 118 by telephone and 27 in person at the outpatient clinic. The mean age of the participants was 63 years (range: 38-84). A total of 47% of patients underwent conservative treatment and 53% underwent surgical treatment (Table I). The prevalence of LHL in participants contacted by phone was 9.3% (11/118) and in person at the outpatient clinic the prevalence was 40.7% (11/27). The overall prevalence of LHL in participants with a RCR was 15.2% (22/145). A higher rate of LHL was found in older patients (*P* = .032). Twelve participants with LHL consented to take part in a semistructured interview. The study population comprised 9 males and 3 females, with a median age of 65 years (range 54-75). Eight participants (67%) underwent surgical treatment, the majority were of Caucasian ethnicity (83%), and over one-half (58%) were cohabiting without resident children. The characteristics of the interview participants are presented in Table II.

**Table I:** Characteristics of participants to determine prevalence of limited health literacy.

Parameters	No limited health literacy (n = 123)	Limited health literacy (n = 22)	*P* value
Gender, *n* (%)			.073∗
Male	71 (58)	17 (77)	
Female	52 (42)	5 (23)	
Age in years, median (range)	62 (38-84)	68 (50-80)	.032†
Type of treatment, *n* (%)			.213∗
Surgical	68 (55)	9 (41)	
Conservative	55 (45)	13 (59)	

∗ Chi-squared test.

† Mann-Whitney test.

**Table II:** Characteristics of interview participants.

Parameters	Total n = 12
Sex, *n* (%)	
Male	9 (75)
Female	3 (25)
Age in years, median (range)	65 (54-75)
Type of treatment, *n* (%)	
Surgical	8 (67)
Conservative	4 (33)
Ethnic background, *n* (%)	
Dutch ethnicity	10 (83)
Non-Dutch ethnicity	2 (17)
Household composition, *n* (%)	
Cohabiting with nonresident children	7 (58)
Living alone without children	2 (17)
Living alone with nonresident children	1 (8)
Cohabiting with resident children	1 (8)
Cohabiting with resident children and caregiving duties	1 (8)
Comorbidity, *n* (%)	
Parkinson	1 (8)
Alzheimer	1 (8)
Ischemic stroke	1 (8)

### Identified challenges

Five themes were identified relating to the challenges participants faced in understanding, finding, and using health information and services during their care process for rotator cuff tears. These themes were: language barriers in health care communication, challenges in finding and appraising health information, limited digital skills, participating in shared medical decision-making, and diverse needs in health information provision. The themes, subthemes, and corresponding open codes were structured in the coding framework presented in Table III.

**Table III:** Code tree of identified challenges.

Open codes	Subthemes	Themes
Difficulty understanding Dutch during medical conversations	Non-native speaker	Language barriers in health care communication
Difficulty fully understanding information from consultations		
Trouble conducting conversations in Dutch independently		
Needs support to understand Dutch medical information		
Lack of multilingual communication options		
Complex words reduce understanding	Use of medical jargon	
Avoiding difficult words improves clarity		
Complex language discourages from reading		
Disengaged when encountering difficult terms		
Feeling overwhelmed by medical jargon		
Limited ability to search for information	Difficulty finding health information	Challenges finding and appraising health information
Uncertainty about where to find information		
Preference for verbal information over independent searching		
Challenges in judging reliability of online sources	Difficulty appraising health information	
Dependence on health care professionals for trusted guidance		
Hospital's online environment is difficult to use	Online information is sometimes difficult to find	Limited digital skills
Varying levels of digital literacy		
Participant does not have an email address		
Difficulty remembering passwords	Trouble learning digital skills	
Unable to manage digital tasks due to cognitive decline		
Desire for active involvement and choice	Varied preferences for involvement in decisions	Participating in shared medical decision-making
Prefers discussing all available treatment options		
Preference to defer to medical authority		
Perception of limited or no real choice		
Stress and memory issues made it hard to ask questions.	Barriers to asking questions and engaging during consultations	
Decision-making affected by limited cognitive abilities		
Overwhelmed during consultations		
Efforts to seek clarity despite challenges		
Varying levels of information-seeking behavior	Variation in capability and confidence	Diverse needs in health information provision
Memory issues, stress, or anxiety limit information processing		
Low confidence in retaining or understanding information		
No need for extra information due to inability to remember		
Brief and clear messages to avoid confusion or anxiety	Preferences in format and delivery of information	
Desire for comprehensive information to avoid independent searching		

#### Language barriers in health care communication

Language barriers were a prominent challenge in participants' experiences with health care communication. These barriers limited their ability to understand medical information. During consultations, non-native speakers experienced difficulties in understanding spoken native language. To understand medical information, participants frequently relied on support from others to interpret and clarify. Participants highlighted the lack of multilingual communication options as a major barrier to access and navigate health care services effectively. Both native and non-native speakers frequently struggled to fully grasp the content of consultations, particularly when health care professionals used complex terms or medical jargon. This use of specialized language led to feelings of confusion and disengagement. Participants described actively avoiding or skipping written materials when confronted with difficult words, as the complexity discouraged further reading.

“When there are a lot of difficult words in a row, I just can't make sense of it, and I stop reading.” (Participant 7, age 56, cohabiting, nonresident children).

#### Challenges in finding and appraising health information

Another key challenge identified was the difficulty participants experienced in finding and evaluating health information. Participants reported they seldom searched for health information themselves. Some lacked interest, while others indicated they did not know where or how to initiate such a search. Even when they did attempt to search for information, they often struggled to determine whether the source was reliable or relevant to their situation. As a result, many participants preferred to rely on their health care professional as their primary and most trusted source of health information, indicating a strong preference for receiving clear, personalized guidance directly from health care professionals rather than navigating information independently.

“I don't really know how to judge what's good information. That's why I don't use the internet much, sometimes the information is good, sometimes it's not, and I can't tell which is which.” (Participant 6, age 56, cohabiting, nonresident children).

#### Limited digital skills

Participants reported difficulties with navigating online health care environments, highlighting a broader challenge related to limited digital skills. Particularly for those with lower levels of digital literacy, hospital websites and patient portals were often described as confusing or unintuitive. Some participants did not have an email address or were unable to remember their login credentials, such as passwords, which further hindered their ability to access digital services. Cognitive decline made it impossible for some participants to complete digital tasks independently. In several cases, this was due to age or health conditions. Tasks affected included booking appointments or accessing test results online. These barriers not only limited access to important health information but also contributed to feelings of frustration and dependency on others for managing healthcare online.

“I prefer information on paper, I've never really been into computers, they've just never interested me.” (Participant 2, age 74, cohabiting, resident children).

#### Participating in shared medical decision-making

Participants expressed mixed experiences with shared decision-making during medical consultations. While some wished to be actively involved in exploring different treatment options, others either felt there was no real choice available or did not feel the need to be part of the decision-making process. Patients felt hesitant or uncertain about asking questions during consultations, especially when cognitive limitations were present or when emotional stress made it difficult to think clearly in the moment. Although some participants made efforts to ask additional questions until they fully understood their situation, others preferred to simply follow the doctor's advice without question, either out of trust or because they found the interaction too overwhelming to engage with more actively.

“I don't feel the need to discuss treatment options; I trust that doctors know what's best, so I'll go with their recommendation.” (Participant 3, age 59, living alone, no children).

Participant: “I would” like to be more involved in the treatment decision, now the decision was made for me." (Participant 5, age 67, cohabiting, nonresident children).

#### Diverse needs in health information provision

The findings also highlighted the wide variation in participants' preferences and abilities regarding receiving and processing health information. Some participants actively sought out information and felt confident in doing so. In contrast, others did not look for information, often due to memory problems, stress, or a belief that they would not retain the information anyway. For some, too much information caused anxiety or confusion, leading them to prefer short, simplified explanations. However, other participants preferred receiving detailed information so they would not need to search for additional answers themselves.

“Important or unimportant things, they all slip away immediately. I prefer explanations to be as brief as possible.” (Participant 10, age 72, cohabiting, nonresident children).

### Proposed solutions

The analysis of proposed solutions revealed several ways to improve health care communication and service delivery to be better aligned with participants' diverse needs and capabilities. The themes, subthemes, and corresponding open codes are structured in the coding framework presented in Table IV. The findings point to a strong desire among participants for more personalized, accessible, and supportive approaches to navigate health information and care.

**Table IV:** Code tree of proposed solutions.

Open codes	Subthemes	Themes
Receives help with online tasks	Digital support	Support needed from environment to manage health
Needs assistance with filling out digital forms		
Prefers in-person over digital communication due to limited digital skills		
Brings partner or family to appointments for memory support	Cognitive and memory support	
Relies on others to remember or explain medical information		
Finds it hard to ask questions or retain information during appointments		
Needs assistance managing appointments	Practical support in daily life	
Accompanying to hospital appointments		
Help with filling out forms		
Asking questions when things are unclear		
Difficulty managing alone after surgery due to lack of care		
Family helps with household tasks and personal care when needed		
Relies on family for help translating Dutch communication	Linguistic support and communication support	
Needs translated written materials or support completing forms		
Prefers a doctor who speaks the same language		
Needs family support to understand healthcare communication		
Prefers receiving information by post	Preferred channels for communication and information	Personalizing health care information
Prefers receiving information by email		
Prefers making appointments in person		
Prefers making appointments by phone		
Preference for written leaflets		
Preference for written leaflets for video formats		
Prefers verbal explanations supported by written materials		
Needs concise and jargon-free information	Information format and content preferences	
Prefers calm and clear communication		
Appreciates visual aids and simple language		
Wants the option to access more detailed information		
Decision aids seen as optional		
Decision aid especially helpful for older adults and non-native speakers		
Prefers questionnaires that show one question at a time		
Needs written information and forms in native language		
Preference for doctor who speaks the same language		

#### Personalizing health care information

A key theme that emerged was the importance of personalizing health care information in both content and delivery, to match individual preferences. Participants showed differing preferences for communication methods, some preferred digital communication, while others opted for mail. Similarly, some favored scheduling appointments in person, whereas others found it more convenient to do so over the phone. There was a clear aversion to the use of medical jargon, with participants favoring calm, clear communication in plain language. The format of information also mattered to many participants, some preferred concise leaflets, others found videos more accessible, and many valued verbal explanations supplemented combined by leaflets and videos. Participants also found visual aids and the option to access more detailed information through links helpful. It is important to make online hospital platforms intuitive and accessible, for example, by building a user-friendly interface and offering content in multiple languages. Decision aids, particularly for older adults and non-native speakers, were welcomed but should be optional. Preferences extended to the structure of digital tools, with some participants favoring questionnaires that present 1 question at a time. Language accessibility was a recurring theme, with participants expressing a need for written information and forms in their native language, and even a preference for doctors who speak the same language. These findings suggest the importance of offering flexible, multilingual, and user-friendly communication strategies tailored to patients' needs and preferences.

“It would be really helpful if the information leaflet was available in more languages. That way, I could read it myself without needing help from my children.” (Participant 12, age 54, cohabiting, resident children).

#### Support needed from environment to manage health

In addition to personalization, participants emphasized the need for support from their environment to effectively manage their health. Digital support was essential for many, with family members often assisting in searching for information, managing online appointments, or completing digital forms. Cognitive and memory support also played a crucial role, some participants brought a partner to appointments to help remember key points, while others relied on family members to ask follow-up questions or to clarify what had been discussed. Linguistic support was another vital area, particularly for those with limited Dutch proficiency. Participants depended on relatives, most often children, for help with interpreting or completing forms or translation during appointments. A lack of sufficient support after surgery was reported to cause difficulties during recovery. It was considered important to clearly discuss beforehand what participants will and will not be able to do after the surgery, in order to determine whether support will be needed at home and what kind of help should be arranged.

“After my shoulder surgery, it was really tough. I couldn't get dressed or manage the house on my own. I didn't have proper home care, and I didn't realize how much help I would need. My daughter helped when she could, but there was no one else around. That time was really difficult.” (Participant 11, age 55, living alone, nonresident children).

**Figure 1:**
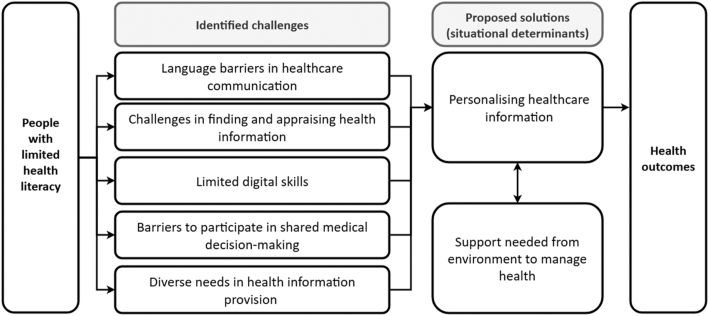
Visualisation of identified challenges and proposed solutions.

Our findings, as visualized in Figure 1, presents how the challenges and proposed solutions identified by participants during the interviews are interconnected. The challenges include language barriers in health care communication, limited digital skills, difficulties in locating and appraising health information, and obstacles to shared decision-making. Participants also reflected on what could help address these difficulties, leading to the shared proposal of personalizing health care information, both the content and the accessibility, as a key solution. The greater the extent to which information is tailored to patients' individual needs, the less reliance there tends to be on support from family members or other informal caregivers, which can promote greater independence in managing health. However, for some individuals, personalization alone will not be sufficient, and ongoing support from their social environment will remain essential. The level of support required will therefore vary according to individual circumstances.

## Discussion

The aim of this study was to examine the 1) prevalence of LHL among patients with rotator cuff tears, 2) challenges they face in managing health information, and 3) potential strategies to address these challenges. The prevalence of LHL in participants contacted by phone was 9.3% and in person at the outpatient clinic the prevalence was 40.7%. Identified challenges included: language barriers in health care communication, difficulties in finding and appraising health information, limited digital skills, barriers in shared decision-making, and diverse needs regarding health information provision, including preferred channels for communication and information as well as specific preferences for the format and content of that information and the method it can be accessed through. Proposed solutions included personalizing health care information, access to the information and when needed additional support from their social environment to manage health.

Our study revealed striking differences in the prevalence of LHL depending on the method of patient recruitment. Initially, we contacted patients who had previously provided informed consent to participate in research. Among this group, we observed a prevalence of LHL of only 9.3%, which was substantially lower than expected based on existing literature.^16^ Given this unexpectedly low prevalence, we considered the possibility of selection bias within this group. It is well-recognized that individuals with LHL are less likely to participate in scientific research for a variety of reasons, including difficulties in understanding study information, mistrust, or feeling overwhelmed by study procedures.^14^ We subsequently approached patients directly at the outpatient clinic in person to reach to desired sample size. This method yielded a prevalence of 40.7% LHL, which aligns more closely with earlier studies.^16^ Therefore, we expect that the actual prevalence is likely to be closer to 40.7%. These findings underscore the importance of appropriate recruitment strategy in studies and highlight how standard research recruitment processes may inadvertently exclude those with LHL or lower socioeconomic status.

The barriers identified in this study, include language difficulties in health care communication, challenges in finding, accessing and appraising health information, limited digital skills, and obstacles to shared decision-making. Previous studies have similarly highlighted that individuals with LHL frequently encounter problems in understanding medical information, and engaging effectively in consultations.^31^ Our findings also align with more recent work emphasizing the impact of limited digital health literacy, particularly as health care increasingly relies on digital tools for information delivery and patient interaction.^13^ Notably, our participants frequently reported depending on family members for support with digital tasks, reflecting how digitalization of health care, such as appointment scheduling or receiving information, can amplify existing barriers when patients lack adequate digital skills. While most of the identified barriers correspond with established literature, our study did not reveal some of the issues described in other contexts, such as difficulties navigating the health care system or mistrust towards health care professionals.^6^ A possible explanation is that participants in our study had already successfully accessed care at the orthopedic outpatient clinic, which may suggest fewer difficulties with system navigation and more positive prior health care experiences. It is important to recognize that LHL is not a single, uniform concept but rather a spectrum encompassing multiple domains, as reflected in the Health Literacy Questionnaire.^23^ Although participants often managed to understand information sufficiently to know what actions to take, actively managing their health, including participating in shared decision-making, proved considerably more challenging. This suggests that limitations in one domain, such as comprehension of health information, may impede more complex tasks like health management and engagement in shared decisions, underscoring the interconnected nature of different health literacy domains. This interaction is also described in the integrated model of health literacy,^31^ where the domains of access, understand, appraise, and apply are conceptualized as sequential and interdependent steps. We acknowledge that health literacy is a broader construct, encompassing not only these domains but also contextual aspects such as health care, situational and personal determinants, and health outcomes, as outlined in the integrated model of health literacy.^31^ In this study, however, our focus was deliberately restricted to a subset of these dimensions, which allowed for a more in-depth exploration of the specific challenges under investigation.

Participants proposed solutions including the need for personalized health information adapted to individual preferences regarding language, format, content, and communication channels and the need for social support to manage health. Tailoring information has repeatedly been shown to support comprehension, foster patient engagement, and reduce disparities associated with LHL.^29^ Although tailoring information is possible, it remains challenging. Health care professionals often, unintentionally, still use complex language, even in patient education materials published in peer-reviewed journals.^28^ Bhatt et al^1^ observed that, despite 3 decades of attention, the average readability level of patient education materials on dyslipidemia has remained largely unchanged, consistently aligning with an 11th-grade reading level. Strategies to improve readability are including patient advocates, plain language experts, or focus groups before patient education materials are published.^1^ Other design elements, including layout, typography, and the integration of visuals, play an equally important role in making educational content effective.^8^ Supplementing written information with easier formats such as videos or implementing more advanced technology such as the use of patient-facing chatbots in patients’ native language, next to social support plays a vital role in enabling individuals with LHL to independently manage their health.^12^^,^^18^^,^^21^^,^^35^ In any case these different methods of providing information should be easily accessible, in terms of digital skills, language and level of literacy. Our study did not generate suggestions for broader systemic or structural interventions, such as simplifying health care processes or improving organizational health literacy, which have been advocated in the literature as critical strategies to reduce health literacy-related inequalities.^7^ This may reflect the individual-level focus of our interviews or the participants' limited awareness of the potential for system-level change.

### Strengths and limitations

A key strength of this study is its mixed-methods design, combining quantitative and qualitative approaches. This provided both an estimate of the prevalence of LHL in our outpatient population and valuable insights into patients' lived experiences. The qualitative component ensured that patients' voices were heard, revealing challenges and preferences that may not emerge from surveys alone. The iterative nature of the interviews allowed us to explore emerging themes in depth. However, some limitations must be acknowledged. Health literacy is multidimensional, and it is challenging to capture the full spectrum of reduced health literacy within a single study sample. We addressed this by using theoretical sampling to include participants with a migration background and female gender. Despite our efforts to ensure diversity, some subgroups were under-represented in the sample, this includes the absence of individuals with congenital conditions, as well as a relatively low number of participants with a migration background and female participants. Our results should be interpreted within the context described in our method section. Consequently, the specific challenges and support needs of some subgroups may not be fully reflected in our findings. This study sample was drawn from Northern and Middle Limburg, a region in the Netherlands marked by a notably fast ageing population, higher proportions of older adults, and socioeconomic indicators that are less favorable compared to national averages.^17^ These regional characteristics may have influenced our results, and limit generalizability to other regions or countries may be limited. For meaningful application, it is important to consider whether the demographic and socioeconomic context of other settings is comparable to that of Northern and Middle Limburg.

### Implications for practice and future research

Our study highlights the importance of tailoring health information to the individual patient with a rotator cuff tear. However, this is only possible when health care providers are able to actively explore and understand each patient's specific needs and preferences, a process in which the health care provider plays a key role. Therefore, health care providers should acquire skills and awareness to identify patients' individual needs, preferences, and capacities. Technology, such as chatbots in patients’ native language, can play a supportive role, provided they enhance accessibility.

Practical strategies for personalizing information include checking for understanding, for example through the teach-back method, which helps confirm whether information is truly understood.^9^^,^^32^ Tools like decision aids, such as visualized option grids for rotator cuff tear treatment options, can support shared decision-making by presenting choices in a structured, accessible way.^22^ In the Netherlands, patient-informed decision aids are developed, exemplifying tools that facilitate shared decision-making.^22^^,^^25^ Information should be delivered in plain language, free from jargon, to make it accessible to everyone, including people with limited reading skills. For example, health care professionals should use terms like shoulder muscles and tendons or cup of the shoulder joint, rather than rotator cuff or glenoid. Adopting a universal precautions approach, assuming that all individuals may have difficulty understanding health information, reinforces the practical value of clear communication.^10^ This approach helps reduce stigma, ensures equity, and acknowledges that health literacy can vary by context and moment.^10^

Moreover, co-developing educational materials together with patients with rotator cuff tears can help ensure that content is relevant, understandable, and aligned with diverse literacy levels. In the Netherlands, ‘Begrijp je Lichaam’ provides patient co-designed visuals and texts for comprehension of the body and also specifically for the shoulder.^24^ Multilingual information and varied delivery channels, ranging from face-to-face conversations to printed leaflets to digital applications, remain essential, but should always be matched to individual abilities and preferences. Digital solutions should be accessible, offer adjustable complexity, and include nondigital alternatives for those less comfortable with technology. Future research should explore how best to support health care providers in developing these competencies and how co-creation with patients with rotator cuff tears can enhance the accessibility and effectiveness of health information.

## Conclusion

This study adds to the evidence that LHL is common and associated with multiple challenges in health care engagement. Addressing these challenges through personalized, accessible communication strategies, in combination with support from their social environment, should be a priority for health care systems aiming to reduce inequalities and improve patient outcomes.

## Disclaimers

Funding: No funding was disclosed by the authors.

Conflicts of interest: Frederik Heerspink reports that he is paid consultant for Arthrex. The other authors, their immediate families, and any research foundation with which they are affiliated have not received any financial payments or other benefits from any commercial entity related to the subject of this article.
